# Assessment of chemical, ultrasonic, diode laser, and Er:YAG laser application on debonding of ceramic brackets

**DOI:** 10.1186/s12903-022-02111-7

**Published:** 2022-03-19

**Authors:** Ahmed S. Khalil, Nazla M. Tamish, Ahmed R. Elkalza

**Affiliations:** Department of Orthodontics, Faculty of Dentistry, Alexandria University, Champollion St., Azarita, P. O. Box: 21521, Alexandria, Egypt

**Keywords:** Ceramic brackets, Debonding, SBS, ARI

## Abstract

**Background:**

Risk of enamel damage that often accompanies ceramic brackets debonding raises the demand of finding an optimal method for debonding of them without adverse effects. Different techniques were proposed in an attempt to facilitate their debonding. Comparison of these techniques is crucial. The aim of this study was to evaluate and compare different techniques for debonding of ceramic brackets in terms of shear bond strength and adhesive remnant index.

**Materials and methods:**

A total of 100 extracted premolars were randomly allocated into 5 groups. Ceramic brackets were then bonded to teeth using light cure composite resin. Among test groups; group I: served as control, group II: chemical aided debonding via peppermint oil, group III: ultrasonic aided debonding, group IV: diode laser aided debonding, and group V: Er:YAG laser aided debonding. Brackets were shear tested using universal testing machine followed by ARI assessment and evaluation of enamel microstructure was performed using scanning electron microscopy.

**Results:**

A significantly lower shear bond strength was found in ultrasonic, diode, and Er:YAG laser groups. However, no significant difference was found in the chemical group. A significantly higher adhesive remnant index was found solely in Er:YAG laser group with minimal enamel microstructure alterations.

**Conclusions:**

Er:YAG laser is a promising tool in debonding ceramic brackets. Ultrasonic and diode laser significantly reduced shear bond strength. Yet, adhesive remnant index in both groups revealed no difference. Chemical aided debonding had little effect and hence, it cannot be recommended without further development.

## Introduction

The advent of ceramic brackets in orthodontics three decades ago was a consequence of the increased number of adult patients seeking orthodontic treatment with less visible appliances [1]. Despite being superior in esthetics, ceramic brackets exhibited higher bond strength and lower fracture toughness in comparison to metal brackets, thus inducing challenges during debonding including enamel tear outs, minute fractures, and cracks [2–4].

Throughout history, attempts have been made to overcome difficulties encountered during debonding, decrease patient discomfort, and keep the bond failure site confined to bracket-adhesive interface. Therefore, broad diversity in terms of debonding techniques were suggested [5–7]. The use of electrothermal debonding technique was proposed for ceramic brackets debonding with controlled heat application [8, 9]. This method resulted in meaningful thermal softening of the adhesive, allowing easier debonding without immoderate force [10]. However, it raised the concern about possible pulpal injury [11]. Custom made especially designed pliers have been introduced with the idea of application of squeezing force [12]. Yet, patient discomfort and enamel damage remained inevitable [13, 14].

On the contrary, literature reported the safety of debonding ceramic brackets using ultrasonic technique [15]. In essence, using ultrasonic tips were claimed to be cost-effective, as the tips used for debonding of ceramic brackets could be used later for removing adhesive remnants [16, 17]. Yet, the significant increase in debonding time remained one of the shortcomings [15].

Various chemical agents have been used for debonding of ceramic brackets with the idea of reducing the required debonding force and hence facilitating removal of the brackets [18]. Application of peppermint oil prior to debonding of ceramic brackets yielded contradictory results; with some reported promising results [18] and others showed no statistically significant difference compared to control [19]. Other authors claimed that peppermint oil altered the site of bond failure and eventually reduced the risk of enamel damage [20]. Inconsistent results with different application time and adhesive resin types were reported, necessitating further investigations [18–20].

Laser irradiation of ceramic brackets have been evaluated in several studies. For instance, carbon dioxide laser (CO_2_) [21–23], ytterbium fiber laser, neodymium-doped yttrium–aluminium garnet (Nd:YAG) [24–26], Erbium, chromium-doped yttrium, scandium, gallium and garnet (Er,Cr:YSGG) [26–28], erbium-doped yttrium aluminum garnet (Er:YAG) [29–31], and diode laser [32–35] were investigated. Mode of action of lasers was reported to be via thermal ablation, photoablation, or thermal softening [29, 30]. Laser aided debonding raised the concern of potential pulp injury as a consequence of raised intrapulpal temperature [32–35]. No statistically significant difference was reported when continuous and pulsed mode were compared [36]. Yet, super pulse yielded superior results in comparison to normal pulse [23, 32].

Laser aided debonding of ceramic brackets have proved its efficiency [35]. With regard to the thermal effect that is often accompanied with laser irradiation, Er:YAG showed success over Nd:YAG and CO_2_ lasers [21]. In fact, Er:YAG revealed the ability to be directly absorbed by the adhesive resin without detrimental consequences on the pulpal tissues [37, 38]. Given the relatively compact size and low weight of diode laser, using it for aided debonding of ceramic brackets would be a privilege [39].

Great concern should be made on reduction of the adverse effects that is often concomitant with ceramic brackets debonding. Yet, there is scarcity of studies with the aim of investigation and comparison of ultrasonic, chemical, and laser aided debonding of ceramic brackets to a control group. Finding an optimal method for debonding ceramic brackets without destructive effects on the enamel is of utmost importance. This would provide great insight for orthodontists on how to reduce patient discomfort that usually accompanies ceramic brackets debonding.

The aim of this study was to evaluate and compare the effects of chemical agent (peppermint oil), ultrasonic instrumentation, diode laser, and Er:YAG laser application on debonding of ceramic brackets in terms of shear bond strength (SBS) and adhesive remnant index (ARI).

## Methods

The study was approved by the institutional review board at the Faculty of Dentistry, Alexandria University (IRB:00010556–IORG:0008839). Informed consent was obtained from all subjects or legal guardians. All the methods were carried out in accordance with CRIS guidelines and regulations. This randomized controlled in vitro study was conducted at Alexandria and Ain Shams University.

### Sample preparation and intervention

**Fig. 1:**
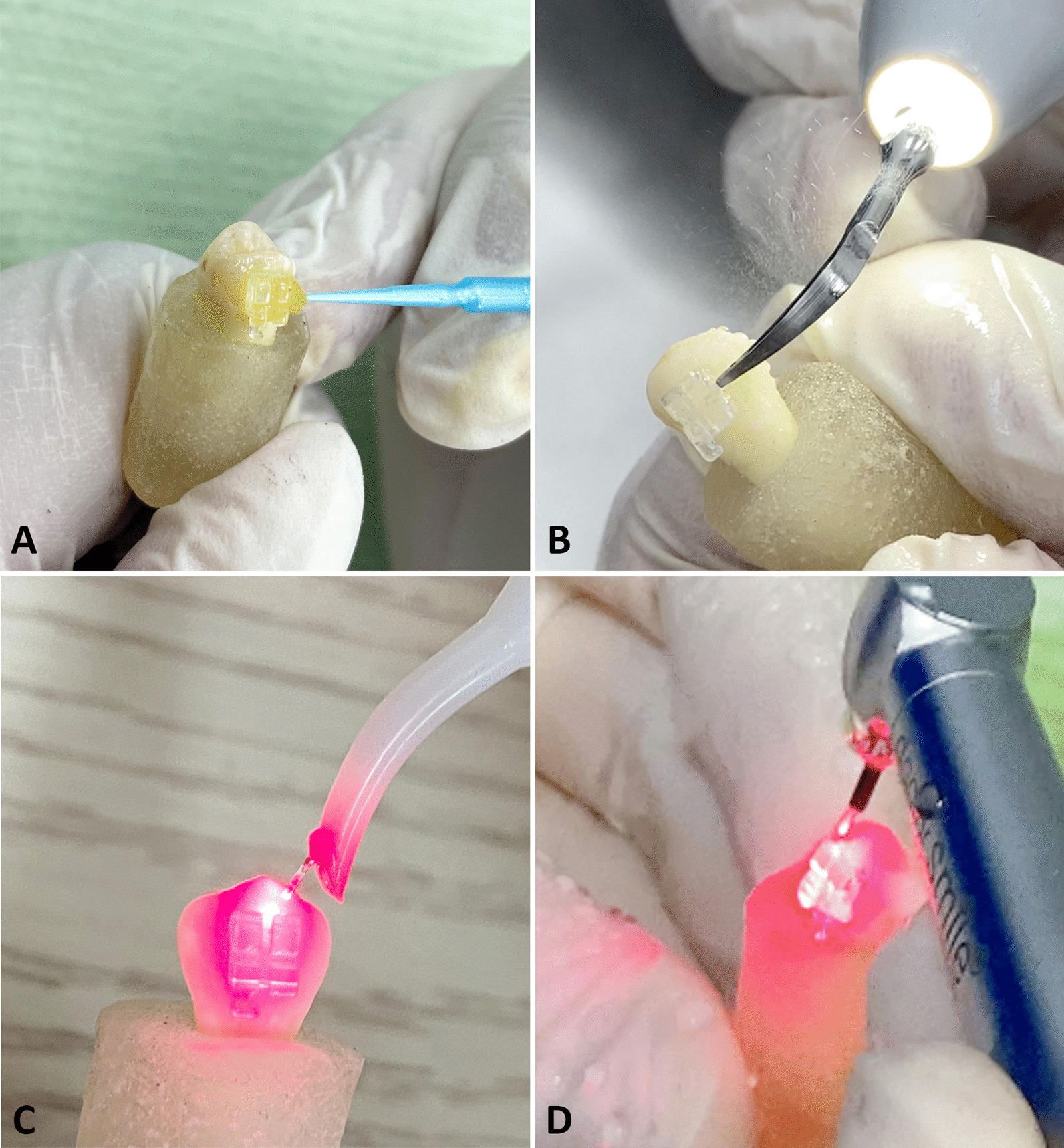
Close-up view of the techniques used **A** Peppermint oil application, **B** Ultrasonic application, **C** Diode laser application, **D** Er:YAG laser application

Sample size estimation was calculated using power and sample size calculation computer software (Epi-Info 7 software, Atlanta, GA, USA). At α = 0.05 and with a power of 0.95, a minimum of 15 teeth per group was required [30]. In order to cater for any damage during the study, 20 premolars per group was used. A total of 100 sound human premolars extracted for orthodontic reasons with intact buccal surface were collected. Patients or their local guardian were informed then signed written consent to allow the use of the teeth. Teeth with carious lesion, restoration, fracture, visible cracks, or hypoplastic lesions were excluded. Teeth were cleaned with tap water and then stored in 0.9% isotonic saline solution. This was followed by random allocation using Random Allocation Software (Version 1.0) [32]. into one of the 5 groups. The buccal surface of the teeth was polished using rubber cup with non-fluoridated oil-free pumice and water, then rinsed and dried with oil/moisture-free air spray. Thereafter, etching of the buccal surface of the teeth was done using 37% phosphoric acid (Denfil, Vericom, South Korea) for 30 s, rinsed thoroughly with water spray for 20 s, and then dried with oil/moisture-free air spray until the enamel had chalky white appearance. In group I, monocrystalline ceramic brackets (Perfect Clear, Hubit, South Korea) were bonded to the center of the buccal surface using the one step GC Ortho Connect adhesive (GC Ortho Connect, GC Orthodontics, Germany) that incorporates the primer into the paste, then firmly pressed, subjected to a 300 g compressive force using a force gauge (Morelli, SP, Brazil) and excess adhesive was removed with a sharp explorer. The adhesive was then light cured with a LED curing light (True dent, Guangzhou, China) for 20 s. After the bonding procedure, teeth were stored in a distilled water for 24 h. The roots of the teeth were then embedded in self cure acrylic resin blocks leaving the crown exposed. In group II, teeth were bonded and then mounted using the same technique employed for group I. Peppermint oil (Peppermint Essential Oil, Areej, Egypt) was applied on the mesial, distal, occlusal, and gingival surface of the brackets for 10 min. (Fig. 1A) In group III, ultrasonic tip (Woodpecker, Guilin, China) with full power was applied as close as possible to the bracket-tooth interface for 12 s: 3 s on each of the mesial, distal, occlusal, and gingival aspects, with sweeping motion in each direction. (Fig. 1B) In group IV, diode laser (Simpler, Doctor Smile, Italy) with continuous mode at a power of 4 W with a wavelength of 980 nm and 300 µm tip diameter, was applied as close as possible to the bracket-tooth interface for 12 s: 3 s on each of the mesial, distal, occlusal, and gingival aspects, with sweeping motion in each direction. (Fig. 1C) In group V, Er:YAG laser (Pluser, Doctor Smile, Italy) at a power of 4 W with a wavelength of 2940 nm, 1 mm tip diameter, 400 mJ energy density, 100 µs pulse duration,10 Hz frequency, 60% water, and 60% air, was applied at the bracket-tooth interface for 12 s: 3 s on each of the mesial, distal, occlusal, and gingival aspects, with sweeping motion in each direction. (Fig. 1D).

### Outcome assessment

**Fig. 2:**
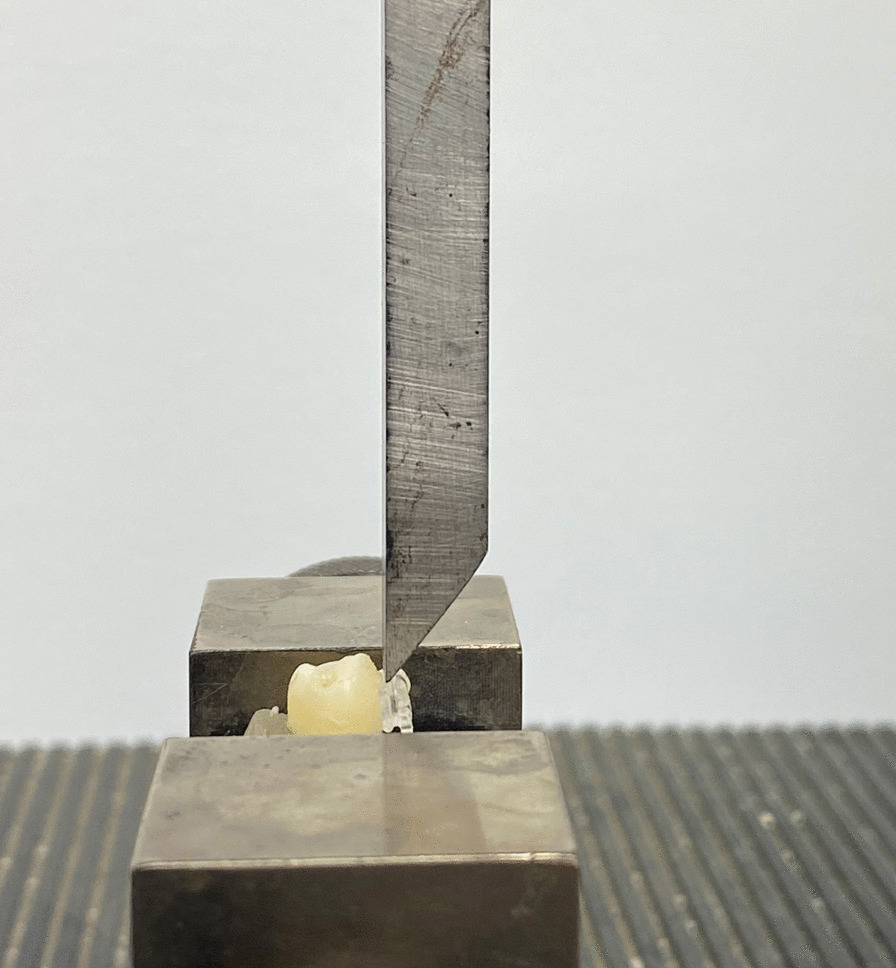
Shear bonding strength test of a sample- lateral view

SBS was tested for each bracket in all the 5 groups using a universal testing machine (LIoyd Instruments Ltd, United Kingdom) through the application of an occlusogingival load with a crosshead speed of 1 mm/min. (Fig. 2) The load at which failure occurred, was recorded for each sample. To express the bond strength in megapascals (MPa), failure load was divided by the bracket base area.

**Fig. 3:**
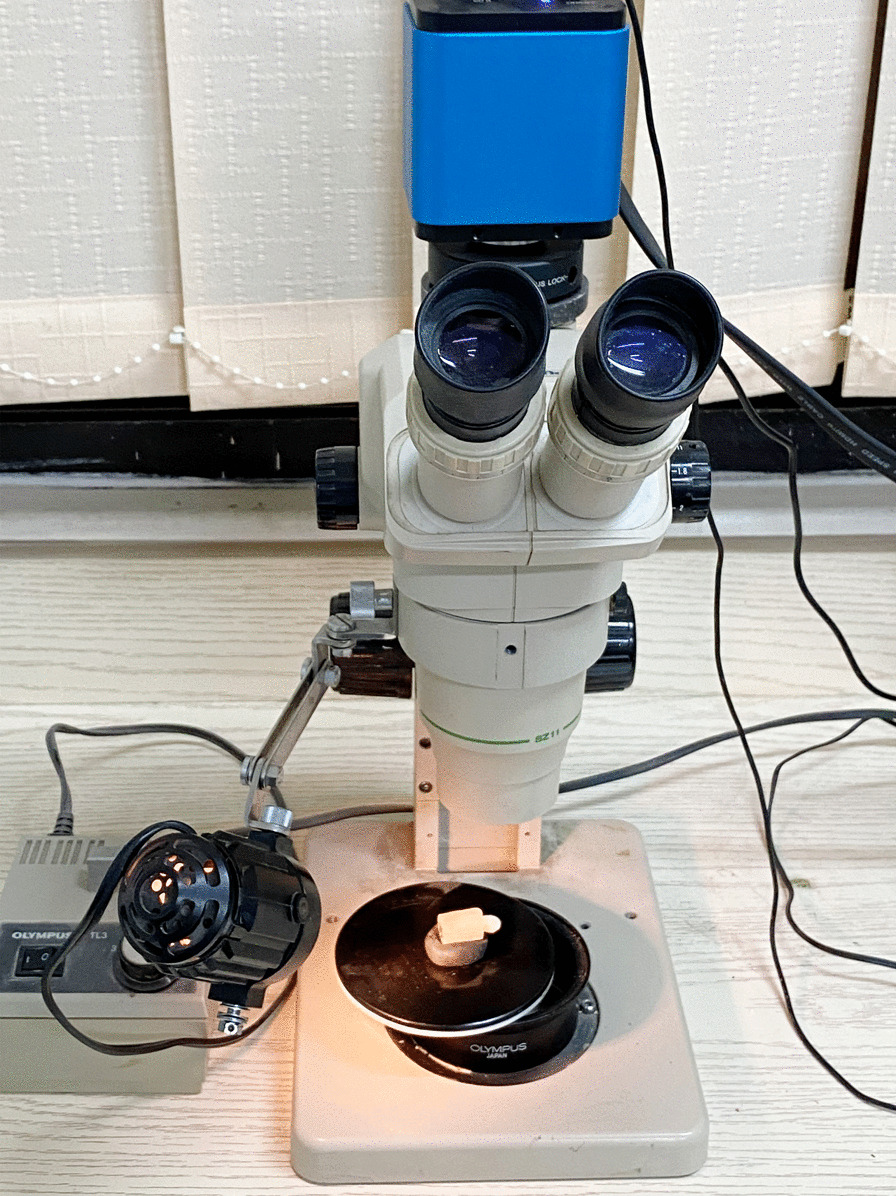
Stereomicroscope

ARI was determined after bracket removal using stereomicroscope (Olympus, Tokyo, Japan) at × 20 magnification. (Fig. 3) ARI scores ranged from 0 to 3 as follows:

- Score 0—no adhesive remaining on the tooth surface.
- Score 1—less than half of the adhesive remaining on the tooth surface.
- Score 2—more than half of the adhesive remaining on the tooth surface.
- Score 3—all the adhesive remaining on the tooth surface.

Intra-examiner reliability was tested by rescoring of the specimens after 2 weeks following the initial scoring. Kappa test exhibited very good intra-examiner reliability.

**Fig. 4:**
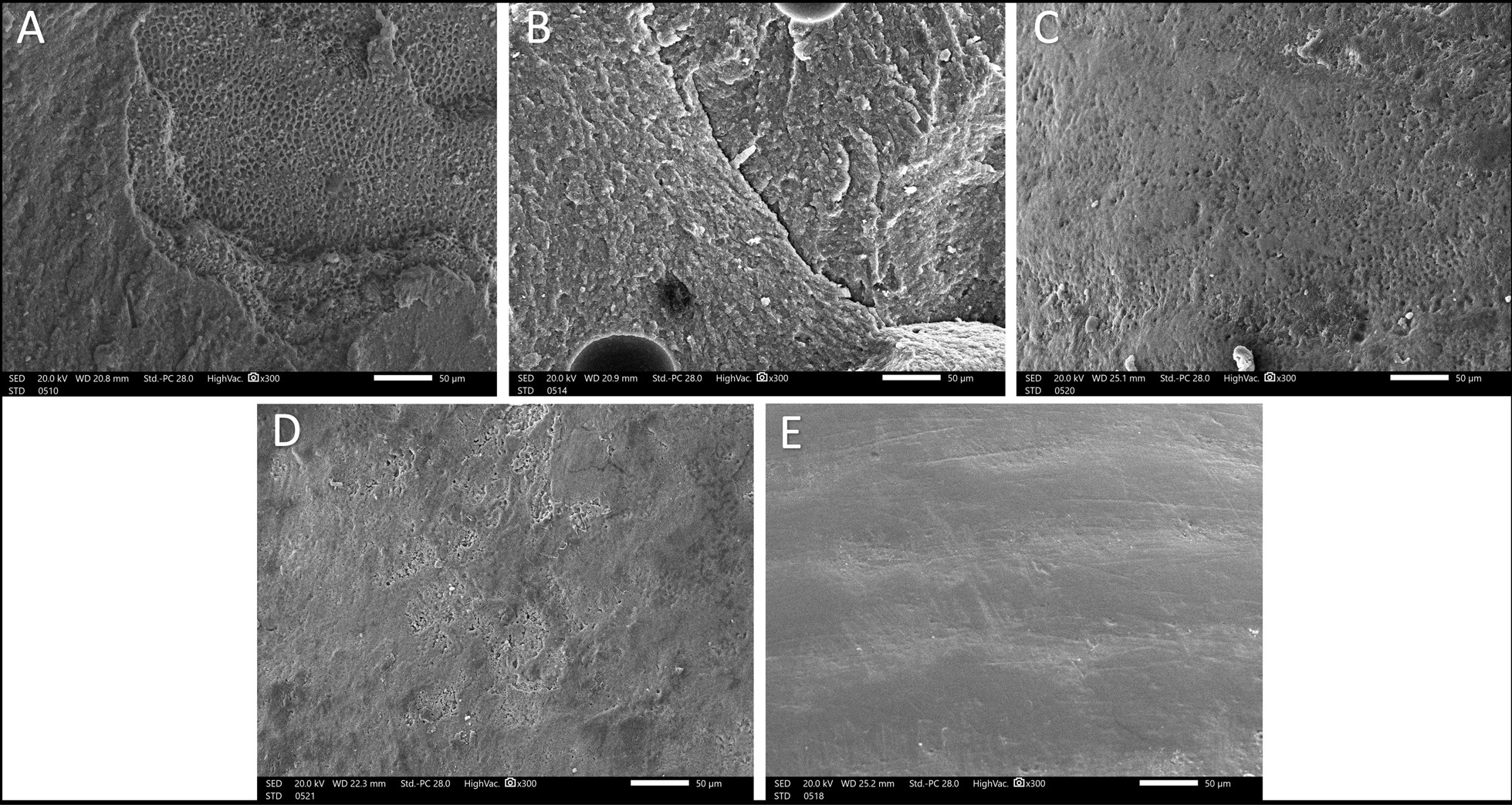
SEM images; **A** group I, **B** group II, **C** group III, **D** group IV, **E** group V (original magnification × 300)

**Fig. 5:**
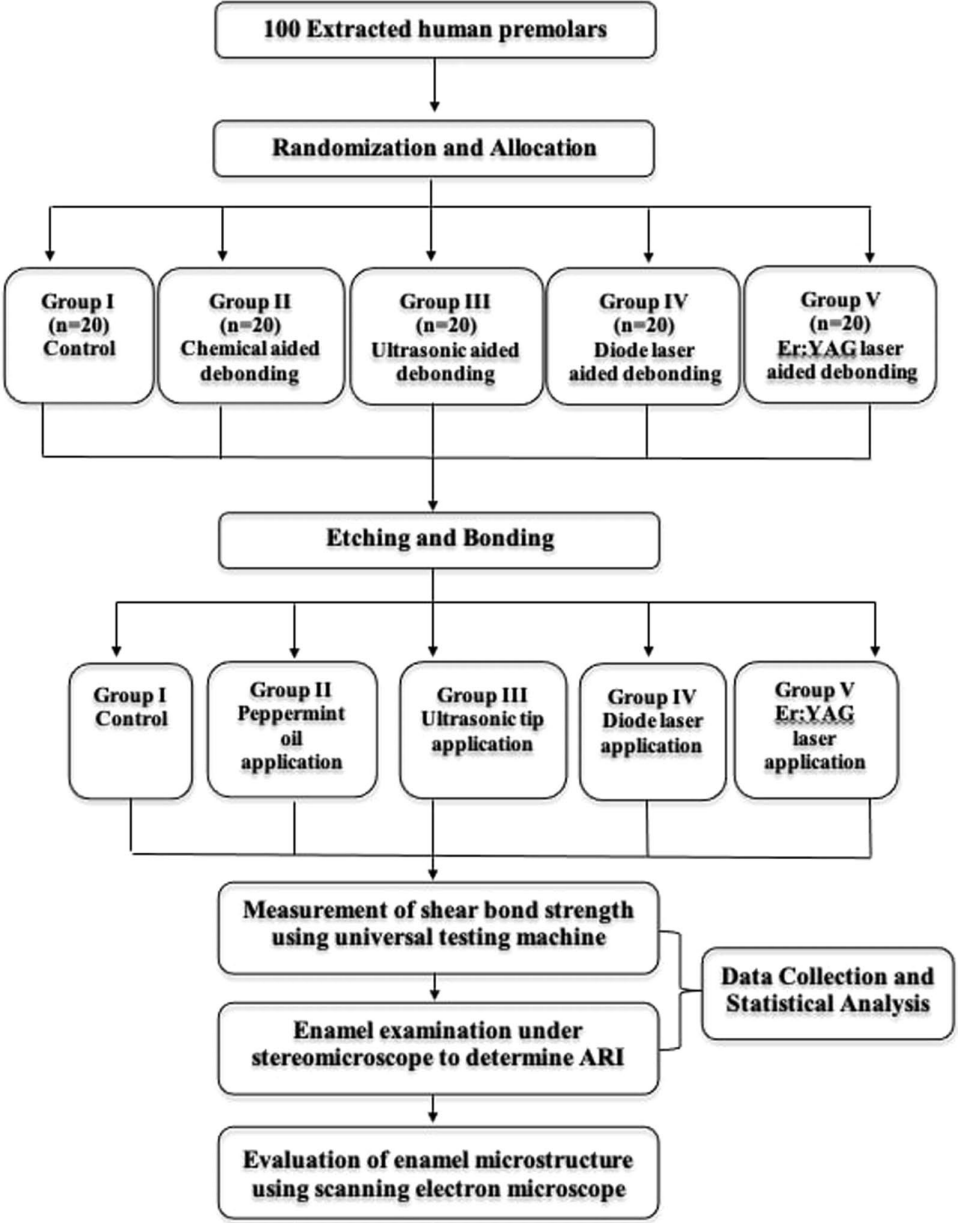
Flow chart summarizing the applied methodology

Enamel microstructure of specimen was evaluated using scanning electron microscopy (SEM) (Jeol JSM-IT200, Tokyo, Japan). (Fig. 4) A flow chart showing the applied methodology is shown in Fig. 5.

### Statistical analysis

SBS values were described using range (minimum and maximum), mean, standard deviation, median and interquartile range. Frequencies and percentages were calculated for ARI. F-test (ANOVA) was used for SBS to compare between more than two groups, and Post Hoc test (Tukey) for pairwise comparisons. Comparison between different groups was done using Chi-square test for ARI. Correction for chi-square when more than 20% of the cells have expected count less than 5, was done using Monte Carlo correction. Significance of the obtained results was judged at the 5% level (*P* ≤ 0.05). Data were analyzed using IBM SPSS software package version 20.0.

## Results

### Shear bond strength

**Fig. 6:**
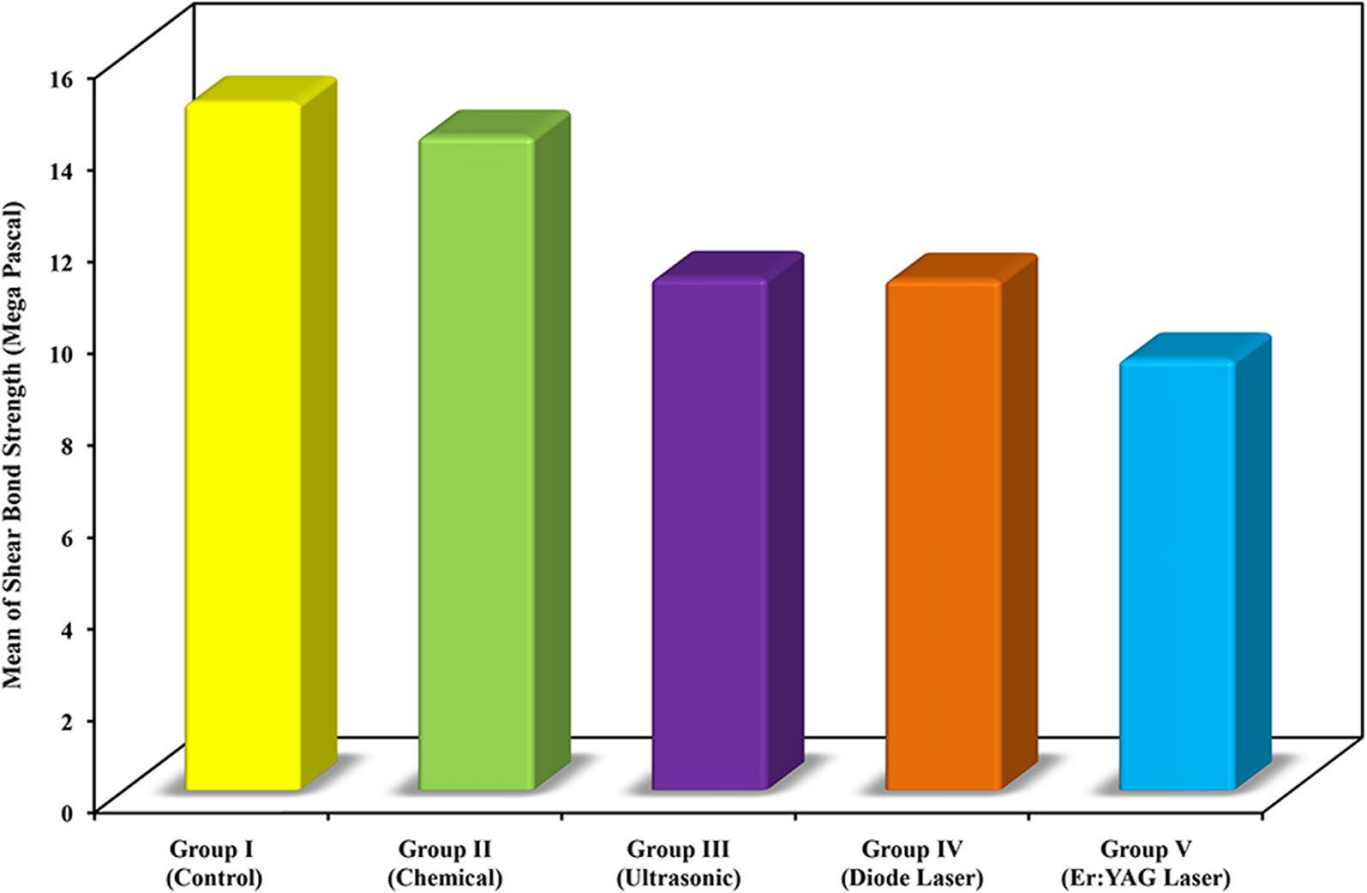
Graphical comparison between the five groups according to mean SBS

Descriptive analysis and analytical statistics of SBS of the five groups are depicted in Table 1. Graphical comparison between the five groups according to mean SBS is shown in Fig. 6. The results of SBS showed a statistically significant difference (*P* = 0.0002). A statistically significant less SBS was found in group III (*P* = 0.039), group IV (*P* = 0.035), and group V (*P* = 0.001) compared to group I (control group). A statistically significantly less SBS was found in group V (*P* = 0.004) compared to group II. There were no other significant differences between the groups with regard to SBS.

**Table 1:** Comparison between the five groups according to shear bond strength

Shear bond strength (mega Pascal)	Group I (control) (n = 20)	Group II (chemical aided debonding) (n = 20)	Group III (ultrasonic aided debonding) (n = 20)	Group IV (diode laser aided debonding) (n = 20)	Group V (Er:YAG laser aided debonding) (n = 20)	F (*p*)
Minimum	4.66	5.81	4.70	5.87	6.94	F = 6.277* (*p* = 0.0002*)
Maximum	26.69	23.77	18.34	17.97	15.20	
Mean	14.99	14.25	11.17	11.13	9.39	
± SD	6.35	4.65	3.51	2.95	2.23	
Median	14.81	14.41	10.54	11.02	8.81	
IQR	10.90–19.72	12.21–16.90	8.73–14.23	9.26–12.95	7.83–11.0	
*p*_0_		0.980	0.039*	0.035*	0.001*	
*p*_1_			0.148	0.138	0.004*	
Sig. bet. grps			*p*_2_ = 1.000, *p*_3_ = 0.664, *p*_4_ = 0.684			

IQR: Inter quartile range; SD: Standard deviation

F: F for ANOVA test, Pairwise comparison bet. each 2 groups was done using Post Hoc Test (Tukey)

*p*: *p* value for comparing between the studied groups

*p*_0_: *p* value for comparing between Control and each other group

*p*_1_: *p* value for comparing between Peppermint oil and each other group

*p*_2_: *p* value for comparing between Ultrasonic and Diode Laser

*p*_3_: *p* value for comparing between Ultrasonic and Er: YAG Laser

*p*_4_: *p* value for comparing between Diode Laser and Er: YAG Laser

^*^Statistically significant at *p* ≤ 0.05

### Adhesive remnant index

**Fig. 7:**
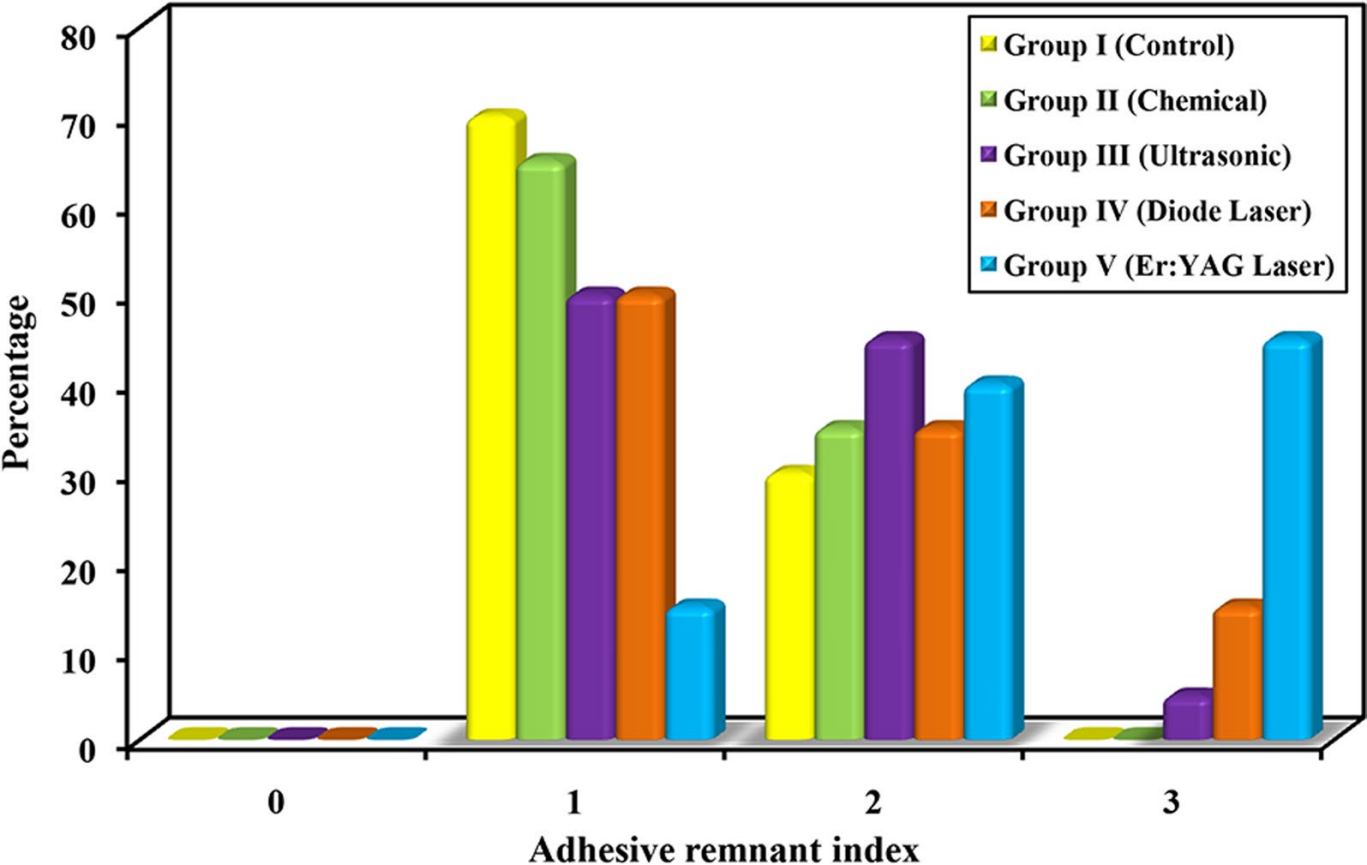
Graphical comparison between the five groups according to ARI

Descriptive analysis and analytical statistics of ARI of the five groups are reported in Table 2. Graphical comparison between the five groups according to ARI is presented in Fig. 7. Statistically significant higher ARI was found solely in group V when compared to group I (*P* = 0.0002), group II (*P* = 0.0002), group III (*P* = 0.006), and group IV (*P* = 0.606). No other significant differences were found between the groups with regard to ARI.

**Table 2:** Comparison between the five groups according to adhesive remnant index

Adhesive remnant index	Group I (Control) (n = 20)		Group II (chemical aided debonding) (n = 20)		Group III (ultrasonic aided debonding) (n = 20)		Group IV (diode laser aided debonding) (n = 20)		Group V (Er:YAG laser aided debonding) (n = 20)		χ^2^ (^MC^p)
	No	%	No	%	No	%	No	%	No	%	
0	0	0.0	0	0.0	0	0.0	0	0.0	0	0.0	25.609 (0.001*)
1	14	70.0	13	65.0	10	50.0	10	50.0	3	15.0	
2	6	30.0	7	35.0	9	45.0	7	35.0	8	40.0	
3	0	0.0	0	0.0	1	5.0	3	15.0	9	45.0	
*p*_0_			0.736		^MC^p = 0.335		^MC^p = 0.184		^MC^p = 0.0002*		
^MC^*p*_1_					0.533		0.241		0.0002*		
Sig. bet. grps					^MC^*p*_2_ = 0.606, *p*_3_ = 0.006*, *p*_4_ = 0.033*						

χ^2^: Chi square test; *MC* Monte Carlo

*p*: *p* value for comparing between the studied groups

*p*_0_: *p* value for comparing between Control and each other group

*p*_1_: *p* value for comparing between Peppermint oil and each other group

*p*_2_: *p* value for comparing between Ultrasonic and Diode Laser

*p*_3_: *p* value for comparing between Ultrasonic and Er: YAG Laser

*p*_4_: *p* value for comparing between Diode Laser and Er: YAG Laser

^*^Statistically significant at *p* ≤ 0.05

SEM revealed extensive mass loss in the form of honey comb alterations, apparent cracks, and erosions in group I and II. Fine voids, facets, scratches, and irregularities in various degrees were observed in in group III and IV. Intact enamel microstructure with little alterations was found in group V (Fig. 5).

## Discussion

The present study aimed to evaluate and compare effects of different techniques for debonding ceramic brackets; chemical (peppermint oil), ultrasonic, diode laser, and Er:YAG laser application to a control group in terms of SBS and ARI.

Various types of chemical agents were investigated with that regard. Acetone and ethanol were tested with different concentrations [40]. The rationale was based on their ability to dissolve the orthodontic adhesive [41]. Nevertheless, neither statistically significant difference in SBS nor ARI were found [42]. Even when longer application time was used, the results gave no support to that hypothesis [43]. On the contrary, eucalyptol in either gel or liquid forms significantly decreased SBS when used in conjunction with metal or ceramic brackets [19, 44]. The present study found no statistically significant difference with regard to SBS and ARI when peppermint oil was used, likewise some previous results [18]. Also, SEM depicted extensive enamel alterations. Longer application period yielded appreciable adhesive softening in a previous study, yet it did not reach statistical significance [19, 45].

Ultrasonic devices have been used for brackets aided debonding and consecutively adhesive removal [19, 46]. The present study found out that ultrasonic application significantly decreased SBS of ceramic brackets. This was in line with Bonetti et al. [18] who demonstrated that ultrasonic instrumentation via both 45 and 0 degree scaler tip angulation significantly decreased SBS values. The results were also in accordance with the two parts study of Bishara and Trulove [5] who found less incidence of bracket failure and decreased likelihood of enamel damage with ultrasonic method. Yet, no statistically significant difference was found in the present study with regard to ARI, similar to a previously reported results by others [17]. SEM also showed various degrees of voids.

Diode laser with its compact size gave it the superiority over other types of lasers. In turn, the use of diode laser for aided debonding of ceramic brackets would be a privilege. Too much debate existed concerning the efficiency of diode laser [5, 15]. This is complicated with the fact that neither a protocol with fixed laser parameters nor clear guidelines exist [17]. The present study found a significant reduction of SBS when diode laser was used. In fact, diode laser yields a coherent radiation with consistent waves which attribute to laser aided debonding via thermal softening of the adhesive. This finding is in agreement with others [32, 47]. Yet, it contradicts the findings of Ivanov [47] who found lack of statistical significance. Multiple factors may have contributed to making the findings of the present study different from it. First, the difference between the bracket pad design. Brackets used in Ivanov study had a patent base with alumina on the center, unlike the ones used in the present study with base coating, employed via double fusing method. Second, the difference in the research design. Power and wavelength of the laser, lasing time, sample size, and type of the adhesive used were all different. On the other hand, ARI analysis showed no significant difference in the present study when diode laser was used, with enamel alterations exhibited under SEM in form of fine scratches. This was in agreement with previous work carried out by others [32–34, 48]. However, Almohaimeed and Abdelhalim [47] and Anand et al. [32, 48, 49] found a significant increase of ARI. Nevertheless, Stein et al. [33] reported a statistically significant reduction. Again, the rationale behind inconsistent findings might be the different applied methodology.

Er:YAG laser yielded promising results when used in prosthodontic field [34]. There is lack of literature on the implication of Er:YAG laser in orthodontics. It is worth mentioning that Er:YAG exhibited less thermal effect than CO_2_ and Nd:YAG laser [50]. Additionally, It is easily absorbed within water containing tissues [51, 52]. In other words, orthodontic adhesive tends to absorb Er:YAG laser energy efficiently. Yet, its relatively large size remains one of the major downsides. The present study demonstrated a statistically significant reduction of SBS when Er:YAG laser was used. This was in agreement with others [39]. Mundethu et al. [53] confirmed the findings and revealed that continuous Er:YAG lasing with no adjunct external force will eventually cause the brackets to jump off the teeth. Sedky and Gutknecht [29, 30, 37, 38] compiled the same protocol with the use of Er,Cr:YSGG laser instead. The mechanism of how SBS decreased is related to the debonding force created from photoablation. The theory behind is explained by the fact that uncured monomer and the water as a components of the adhesive likely absorb laser energy, expand, and thereafter vaporize, giving rise to subsurface pressure and consequently micro-explosions, which cause decomposition of the resin matrix and thereby decrease SBS of the brackets [54]. This phenomenon also elucidated why we primarily chose the one step adhesive that incorporates the primer into the paste. Contrastingly, ARI showed a statistically significant higher values when Er:YAG laser was used. This was consistent with results reported by others [55]. Yet, Dostalova et al. [56] and Sedky and Gutknecht [29, 30, 37] contradicted this finding. This might be attributed to the different laser parameters and different laser types used with the work of Dostalova et al. [57] and Sedky and Gutknecht, respectively. The best case scenario sought would be the increased ARI, which reflected the conservative effect with minimal damage to the enamel [55].

The results of SBS values revealed a significant reduction in all study groups compared to control except the chemical aided debonding, where negligible decrease was observed. In essence, it was believed that a force in the range of 6 to 8 MPa is crucial to debond orthodontic brackets [57]. The only group that showed a merely acceptable values was the Er:YAG laser aided debonding, with a mean SBS of 9.39 MPa. This could make it the technique of choice for debonding ceramic brackets. Despite a significant reduction of SBS in ultrasonic and diode laser aided debonding, mean SBS in these groups was relatively higher than the acceptable range (11.17 and 11.13 MPa, respectively).

ARI analysis was employed via measurement of the amount of remaining adhesive. The findings of the present study exhibited a higher mean of ARI in all test groups compared to control, even though the only group that reached statistical significance was the Er:YAG laser aided debonding, with little enamel microstructure alterations. Again, higher ARI scores markedly reflect the reduction of enamel damage [58, 59]. Yet, increased chair time for the purpose of adhesive removal remained inevitable [60].

## Conclusions

- Er:YAG laser facilitated debonding of ceramic brackets via reduction of SBS and increase of ARI. This may alleviate the risk of enamel damage.
- Ultrasonic and diode laser aided debonding of ceramic brackets significantly decreased SBS. Yet, ARI in both groups revealed no significant difference.
- Chemical aided debonding of ceramic brackets via peppermint oil had little effect on SBS and ARI. Hence, this method cannot be recommended without further development.

## Data Availability

The datasets used during the current study are available from the corresponding author on reasonable request. All data analyzed during this study are included in this published article in the form of tables and figures.

## Abbreviations

Er:YAG: Erbium-doped yttrium aluminum garnet
Er,Cr:YSGG: Erbium, chromium-doped yttrium, scandium, gallium and garnet
Nd:YAG: Neodymium-doped yttrium–aluminium garnet
CO_2_: Carbon dioxide
SBS: Shear bond strength
ARI: Adhesive remnant index
MPa: Megapascal
SEM: Scanning electron microscope
